# Supplementary material for: Correlation between Macular Pigment Optical Density and Neural Thickness and Volume of the Retina
Source: Nutrients. 2020 Mar 25;12(4):888. doi: 10.3390/nu12040888 (PMC7230595; doi:10.3390/nu12040888)
Supplement: Supplementary file 1 [file nutrients-12-00888-s001.pdf]

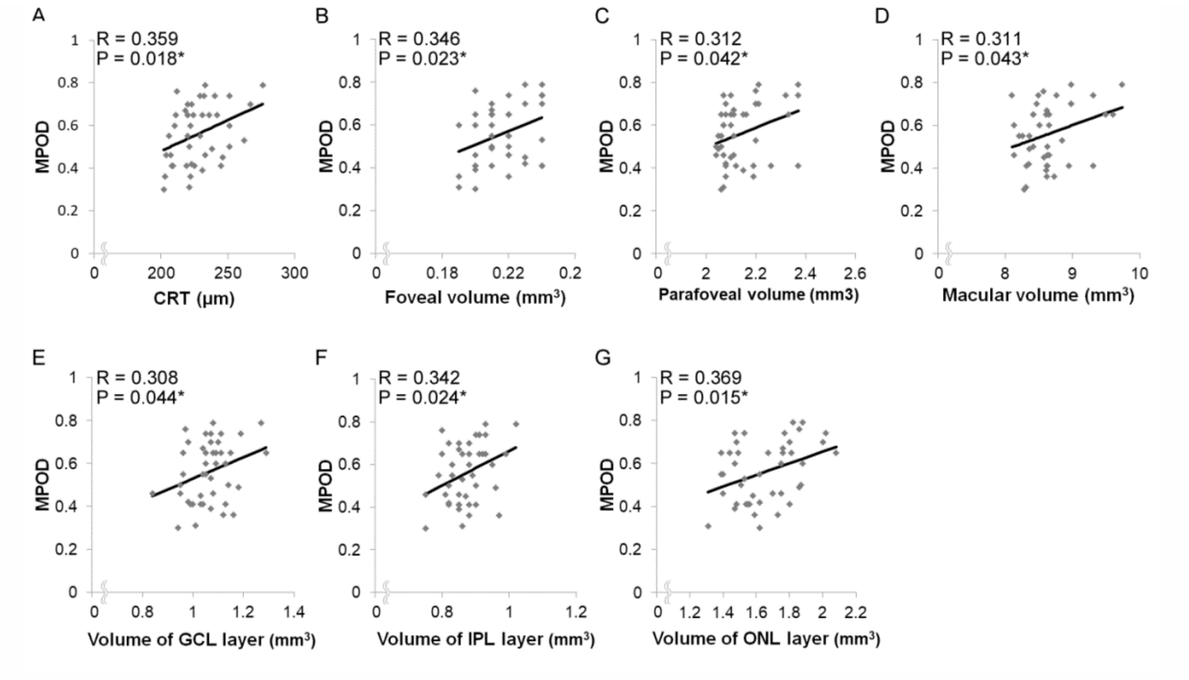

Figure S1. Correlations between MPOD and CRT, foveal, parafoveal, and macular volumes, and GCL, IPL, and ONL volumes of the macular area from the dataset of right eyes which did not have high myopia together with those of the left eye in subjects who had high myopia in the right eye but not in the left eye. MPOD, macular pigment optical density; GCL, ganglion cell layer; IPL, inner plexiform layer; ONL, outer nuclear layer. * $P < 0.05$.
